# A triple-drug combination induces apoptosis in cervical cancer-derived cell lines

**DOI:** 10.3389/fonc.2023.1106667

**Published:** 2023-05-08

**Authors:** Izamary Delgado-Waldo, Carlos Contreras-Romero, Sandra Salazar-Aguilar, João Pessoa, Irma Mitre-Aguilar, Verónica García-Castillo, Carlos Pérez-Plasencia, Nadia Judith Jacobo-Herrera

**Affiliations:** ^1^ Unidad de Bioquímica Guillermo Soberón Acevedo, Instituto de Ciencias Médicas y Nutrición Salvador Zubirán, Tlalpan, Mexico; ^2^ Posgrado en Ciencias Biológicas, Universidad Nacional Autónoma de México. Copilco Universidad, Coyoacán, Mexico; ^3^ Laboratorio de Genómica, Instituto Nacional de Cancerología, Tlalpan, Mexico; ^4^ Laboratorio de Hematopoiesis y Leucemia, Unidad de Investigación, Diferenciación Celular y Cáncer, Facultad de Estudios Superiores Zaragoza, Universidad Nacional Autónoma de México, Iztapalapa, Mexico; ^5^ CNC - Center for Neuroscience and Cell Biology, CIBB - Center for Innovative Biomedicine and Biotechnology, University of Coimbra, Coimbra, Portugal; ^6^ Laboratorio de Genómica Funcional, Unidad de Biomedicina, FES-IZTACALA, Universidad Nacional Autónoma de México, Tlalnepantla, Mexico

**Keywords:** cervical cancer, apoptosis, combinatorial therapy, repurposing drugs, mTOR pathway

## Abstract

**Introduction:**

Cervical cancer is a worldwide health problem due to the number of deaths caused by this neoplasm. In particular, in 2020, 30,000 deaths of this type of tumor were reported in Latin America. Treatments used to manage patients diagnosed in the early stages have excellent results as measured by different clinical outcomes. Existing first-line treatments are not enough to avoid cancer recurrence, progression, or metastasis in locally advanced and advanced stages. Therefore, there is a need to continue with the proposal of new therapies. Drug repositioning is a strategy to explore known medicines as treatments for other diseases. In this scenario, drugs used in other pathologies that have antitumor activity, such as metformin and sodium oxamate, are analyzed.

**Methods:**

In this research, we combined the drugs metformin and sodium oxamate with doxorubicin (named triple therapy or TT) based on their mechanism of action and previous investigation of our group against three CC cell lines.

**Results:**

Through flow cytometry, Western blot, and protein microarray experiments, we found TT-induced apoptosis on HeLa, CaSki, and SiHa through the caspase 3 intrinsic pathway, including the critical proapoptotic proteins BAD, BAX, cytochrome-C, and p21. In addition, mTOR and S6K phosphorylated proteins were inhibited in the three cell lines. Also, we show an anti-migratory activity of the TT, suggesting other targets of the drug combination in the late CC stages.

**Discussion:**

These results, together with our former studies, conclude that TT inhibits the mTOR pathway leading to cell death by apoptosis. Our work provides new evidence of TT against cervical cancer as a promising antineoplastic therapy.

## 1 Introduction

Despite early detection programs, cervical cancer (CC) is a public health problem that still needs to be solved. Although its incidence has decreased by approximately 40% in the last decade, it currently remains the second leading cause of cancer-related deaths in women, accounting for an estimated 270,000 women’s lives annually (1). The use of cervical cancer screening has helped in an early diagnosis of this disease; however, there is still a large population in which the disease is diagnosed in locally advanced stages (2). In such a scenario, the clinical response rates to conventional treatment based on radiation therapy and/or surgery, chemotherapy (3), and immunotherapy (4) are poor. These therapies have systemic side effects on patients (5), high costs (6), and rapid development of drug resistance (7–10), which are some of the limitations that reduce their effectiveness (11).

Warburg’s research in the 1920s proved that tumor cells require a high income of glucose to maintain their extremely demanded metabolism activity independently of the oxygen levels, a process known as the Warburg effect or aerobic glycolysis (12–14). In contrast to the normal cell, the tumor cell produces vast amounts of extracellular lactate *via* aerobic glycolysis (15) and consequently generates several disturbances in other cells and the tumor microenvironment (16), leading to hypoxia, promotion of angiogenesis, and distant metastasis (17, 18). The high rate of glucose consumption allows the tumor cell to obtain biosynthetic precursors necessary for the biosynthesis of nucleotides, proteins, and lipids to achieve increased proliferation, survival, and progression of tumor characteristics (19–23).

Drug repositioning is a current strategy for new anticancer treatments (24) in terms of safety, side effects, and mechanisms of action (25). In this scenario, our research group successfully proved that the combination of the known drugs metformin, doxorubicin, and sodium oxamate (named triple therapy or TT) inhibited tumor growth in cell lines derived from breast and colon cancers and in two different murine models (17, 26, 27). Metformin is widely used to treat type 2 diabetes mellitus (28). Besides it is an antitumor candidate due to its ability to inhibit respiratory-chain complex I (29), activate the protein AMPK, and inhibit the mTOR activation (30). Such events stop cell proliferation and the production of proteins, lipids, and carbohydrates (31–34). The second drug, oxamate, is a competitive inhibitor of lactate dehydrogenase A (LDH-A), an enzyme that catalyzes the pyruvate to lactate in aerobic glycolysis (35–38). Finally, doxorubicin is a well-known drug used to treat several types of cancer (39). It induces cell death in three different ways: topoisomerase II inhibition, inhibition of DNA synthesis, and induction of oxidative stress (39–41).

Previously, we have shown that the TT leads to apoptosis by the upregulation of PARP-1 and the caspase 3 cleavage, as well as mTOR and LDH-A inhibition. Additionally, in murine models, the TT was even more efficient than the doxorubicin treatment and did not present visible toxicity in organs or tissues, and the animals had a longer survival rate than those under the control drug treatment (17, 26, 27). In the present work, we aimed to establish the apoptosis pathway induced by the TT in CC cell lines as a novel proposal to treat CC patients. Hence, we showed that the TT triggered tumor cell death by apoptosis through the caspase 3 intrinsic pathway. These results indicate that the combination of drugs that inhibit different tumor pathways, in this case aerobic glycolysis, nucleic acid synthesis, and complex I of the respiratory chain, has evident antitumor effects. The use of radio- and chemotherapy affects tumor cells with high proliferative rates. Therefore, inhibition of multiple pathways, including aberrant tumor metabolism in cancer therapy, should be considered.

## 2 Materials and methods

### 2.1 Reagents and cell culture

Doxorubicin (Doxolem^®^ RU, 10 mg/5 ml) (Dox), metformin (Met) (1-1dimethylbiguanidine hydrochloride; sc-202000A, Santa Cruz Biotech, USA), and sodium oxamate (Ox) (sc-215880, Santa Cruz Biotech, USA) were diluted with DMEM/F12 medium (GIBCO, USA) supplemented with 2% FBS (ATCC 30-2020), with the following concentrations *in vitro*: 1.5 μM/25 mM/20 mM for dox/met/ox respectively, for 12 h, and 1 μM/20 mM/15 mM for dox/met/ox, respectively, for 24 h.

The human cervical cancer cell lines Hela, SiHa, and CaSki were purchased from the American Type Culture Collection (ATCC). All cells were cultured in DMEM/F12 medium supplemented with 10% FBS and 1% penicillin–streptomycin at 37°C in a humidified atmosphere of 95% air plus 5% CO_2_, as recommended by ATCC.

### 2.2 Cytotoxicity assay

Cell viability was determined using the sulforhodamine (SRB) cell protein stain (42). Cancer cells were seeded in 96‐well plates at a density of 10,000 cells/well and allowed to adhere overnight (24 h). After 24 h of incubation, the cells were treated with different doses of metformin (15, 20, 25, 30, 40 mM), sodium oxamate (10, 15, 20, 25, 30 mM), and doxorubicin (0.5, 1, 2, 3, 4 μM) in combination for 12 and 24 h. Cells were fixed with cold trichloroacetic acid 10% (TCA) (MERCK, USA) at 4°C for 1 h and then washed four times with tap water, followed by staining with 100 µl of 0.5% SRB (S9012-5G Sigma-Aldrich) in 1% acetic acid for 30 min at room temperature (RT). Excess stain was washed four times with 1% acetic acid. The stained cells were resuspended in 200 μl of 10 mM Tris, pH 10. The optical density at 510 nm was determined using an Epoch microplate spectrophotometer (BioTek). All the experiments were done in triplicate.

### 2.3 Apoptosis antibody array and Western blot

The Human Apoptosis Antibody Array Kit (RayBiotech, Inc., Norcross, USA) was used to evaluate the apoptotic protein expression according to the manufacturer’s instructions. The membranes were soaked in blocking buffer at RM for 30 min. Extracted protein (1 mg/ml) from HeLa cells with treatment at 12 h was added into each well containing the membranes and was left overnight at 4°C for incubation. The membrane was added with a biotinylated antibody cocktail and subsequently with HRP-streptavidin for incubation overnight at 4°C for incubation. A total of 500 μl of the detection buffer was pipetted onto the membrane for 5 min at RM. The membranes were transferred and exposed to a chemiluminescence C-DiGit scanner employing the Image Studio (LICOR, USA) software, then they were used to quantify the intensity of each array dot and then normalized to the internal control. All incubations and washes were performed under rotation (~0.5–1 cycle/s).

In order to determine specific proteins, Western blot analysis was done; briefly, cells were collected and lysed with RIPA buffer (Santa Cruz Biotechnology sc-24948) containing protease inhibitors for 30 min on ice and then centrifuged at 13,000*g* at 4°C for 25 min. The supernatant containing total protein was harvested. The concentration was detected using Bradford assay (Bio-Rad) according to the manufacturer’s instructions. Thereafter, 25 μg of proteins was separated by 12% SDS-PAGE and transferred to polyvinylidene difluoride (PVDF) membranes (GE Healthcare, USA) in a semidry Trans-Blot Turbo chamber (Bio-Rad) at 25 V, 1 mA, for 30 min. The membranes were blocked with 5% non-fat milk in TBS containing 0.1% Tween 20 for 2 h. The membrane was incubated with specific primary antibodies Caspase-3 (1:3,000, sc-7148), Caspase-8 (1:2,000, sc-56070), and p21 (1:3,000, sc-397) from Santa Cruz Biotechnology; XIAP (1:1,000, ab28151) from Abcam; and BAX (1:1,000, 12105S) and BAK (1:1000, 5023S) from Cell Signaling Technology, overnight at 4°C on a rocking platform, washed, and then incubated with the corresponding secondary antibodies anti-mouse (1:5,000, sc-23719) and anti-rabbit (1:3,000, sc-2370) for 2 h at room temperature. The control for equal protein loading was assessed using an anti‐β‐actin antibody (1:5,000; sc-47778). The blot was visualized using the SuperSignal West Femto chemiluminescent substrate (Pierce) in the C-DiGit scanner (LICOR)™ employing the Image Studio (LI-COR) software. All the experiments were repeated in triplicate.

### 2.4 Flow cytometry: Annexin V-FITC apoptosis detection

Apoptosis was detected using Annexin V-FITC Apoptosis Detection Kit I (BD 556547) by flow cytometry according to the manufacturer’s instructions. There are two distinct phases in the apoptotic process, termed early and late apoptosis, which can be distinguished with an intracellular staining assay. Annexin V (AV) and propidium iodide (IP) double staining can quantitatively distinguish viable cells from dead cells. Therefore, cells were distinguished into four groups in a scatter plot: viable cells (Annexin V-/IP-) in the lower-left quadrant, early apoptotic cells (Annexin V+/IP-) in the lower-right quadrant, late apoptotic cells together with secondary necrotic cells (Annexin V+/7-AAD+) in the upper-right quadrant, and necrotic cells (Annexin V-/IP+) in the upper-left quadrant. Cancer cells were seeded in six‐well plates at a density of 500,000 cells/well and allowed to adhere overnight (24 h). Then, the cells were treated with the IC_50_ of TT for 12 and 24 h. Briefly, the treated cells were harvested by trypsin, washed with PBS, collected by centrifuging at 1,500*g* for 5 min, mixed with a binding buffer, and incubated with Annexin V-FITC and propidium iodide (PI) for 15 min at RT in the dark. Afterward, labeled cells were counted by flow cytometry within 30 min. All early apoptotic cells (Annexin V–positive, PI-negative), necrotic/late apoptotic cells (double positive), and living cells (double negative) were detected using a FACSCalibur flow cytometer and subsequently analyzed using CellQuest Pro software. All the experiments were performed in triplicate.

### 2.5 Terminal deoxynucleotidyl transferase dUTP nick end labelling assay

DNA damage is one of the main characteristics of apoptosis. The visualization of DNA damage is achieved with terminal deoxynucleotidyl transferase dUTP nick end labeling (TUNEL) staining, which is based on the ability of the enzyme deoxynucleotidyl transferase to catalyze the addition of nucleotide dUTP to the 3′ ends free of fragmented DNA. dUTPs that are FITC-labeled fluoresce when used, and apoptotic cells can be specifically identified. For this purpose, CC cell lines were used and kept in cover slides previously treated with polylysine in six-well plates with a density of 4 × 105 cells in each well with the conditions already mentioned and with 24 h of adherence. They were exposed to the pharmacological combination for 12 and 24 h. After the drug exposure time, the cells were fixed with 4% paraformaldehyde and, through the DeadEnd Fluorometric TUNEL System Cat. G3250 kit, apoptosis was evaluated according to the manufacturer’s instructions. Finally, the cells were observed under a confocal microscope and the resulting images were analyzed using Illustrator software.

### 2.6 Wound healing assay

A wound healing assay was performed to assess migration. An adhesive tape was placed in six-well plates. The HeLa, SiHa, and CaSki cells were seeded (4 × 10^5^ cells per well) and maintained with 2% FBS-supplemented medium to avoid cell proliferation at 37°C, and after 24 h of adhesion, the tape was removed to form the wound. These cells were treated with triple therapy or controls for 12 and 24 h. The cells were monitored at 0, 8, 12, and 24 h.

## 3 Results

### 3.1 TT-induced cytotoxicity against HeLa, SiHa, and CaSki cell lines

**Figure 1:**
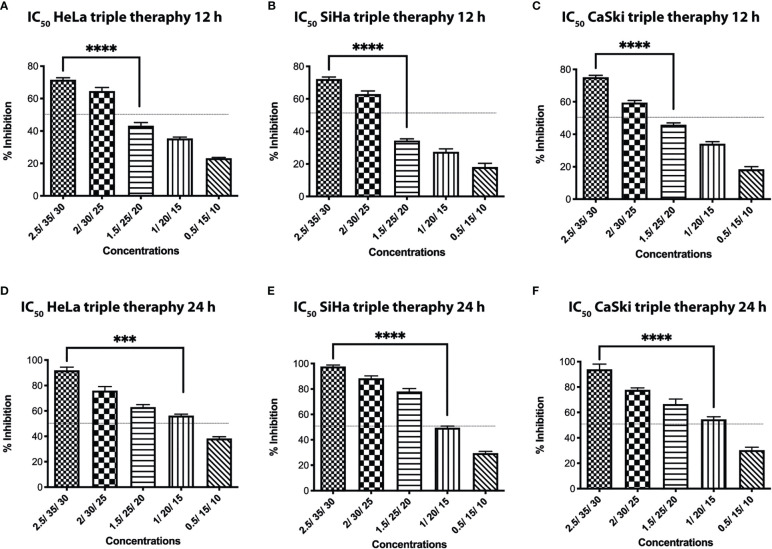
Inhibitory concentration of the TT at 12 **(A–C)** and 24 h **(D–F)** against Hela, SiHa, and CaSki cervical cancer cell lines. For all panels, *P* values were determined by the ANOVA test; ***P ≤ 0.001 ****P ≤ 0.0001.

The triple therapy was evaluated at 12 and 24 h against the three CC cell lines at different concentrations. The time selected for this investigation was based on our previous research on colon and breast cancers (26, 27), where the effect exerted by the TT inducing apoptosis and autophagy starts at short times (4 h). Moreover, in the breast cancer *in vivo* model, tumor reduction with the TT was initiated at 24 h (26). These results provided the timing used in this work. The percentage of cell growth inhibition (IC_50_) depends on the concentration of the drug and is time-dependent (**Figure 1**). The IC_50_ values obtained for the three cell lines were 1.5 μM/25 mM/20 mM for dox/met/ox, respectively, in 12 h, and for 24 h, the IC_50_ values were 1 μM/20 mM/15 mM for dox/met/ox, respectively, and were employed for the other tests.

### 3.2 The apoptosis pathway is triggered by TT in HeLa cells

**Figure 2:**
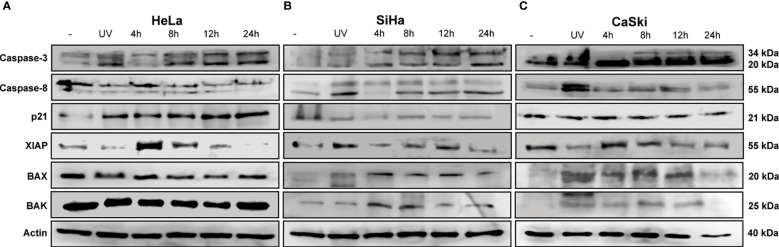
TT-induced intrinsic apoptosis by cleavage of caspase 3. **(A)** HeLa, **(B)** SiHa, and **(C)** CaSki CC cell lines. All cells were treated with the TT at 4, 8, 12, and 24 (h) UV radiation (positive control). The blots are a representative figure of at least three independent experiments.

**Figure 3:**
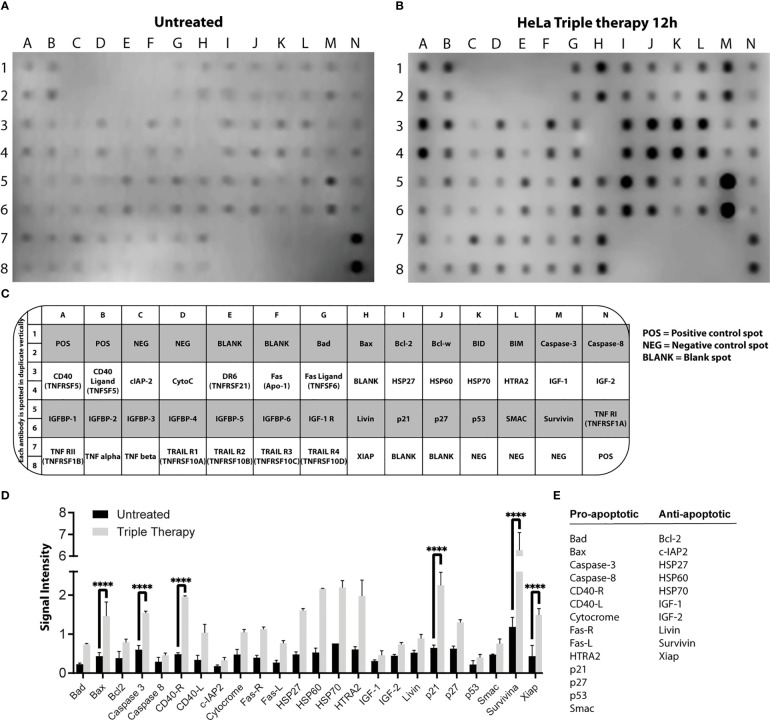
Profile of proteins involved in apoptosis in HeLa cells. Protein extracts of untreated cells **(A)** and treated cells **(B)** with the TT at 12 h of exposure. **(C)** Map array showing the location of apoptosis-related antibodies detected by the Apoptosis Kit. **(D)** Chemiluminescent intensities quantified by densitometry. A positive control was used to normalize the membrane results. **(E)** Proteins were selected and divided into two groups (pro-apoptotic or anti-apoptotic); *P* values were determined by two-way ANOVA test; ****P ≤ 0.0001.

We first characterized the apoptotic response with the triple therapy using a protein profile involved in apoptosis carried out on HeLa cells. For this experiment, we detected the emission of light as a result of a chemical reaction (chemiluminescence) of each point that corresponds to a specific antibody. The microarray map of **Figure 2A** (untreated cells) and **Figure 2B** (cells treated with TT) shows the location of each protein. **Figures 3C, D** depict the overexpression of the pro-apoptotic proteins BAD, BAX, caspase 3, cytochrome C, CD40-R, CD40L, Fas-R, Fas-L, HTRA2, p21, p27, and SMAC. Also, anti-apoptotic proteins such as XIAP, survivin, HSP27, HSP60, and HASP70 were overexpressed after TT treatment for 12 h. The experiment was performed only at 12 h, time enough to demonstrate the apoptosis induction by the triple therapy. The other proteins had no change (**Figure 3D**). The signal intensity of the pro-apoptotic proteins p21, HTRA2, CD40-R, caspase-3, and Bax is remarkable (**Figure 3D**).

The next step was to corroborate the results obtained in the microarray by WB. Proteins involved in apoptosis were evaluated in the three CC cell lines. The IC_50_ values used were those detected for each time frame, as indicated in the Materials and Methods section. **Figures 2A–C** shows that the activity of caspase 3 is time-dependent, increasing to 24 h in all CC cell lines treated with TT. Such results could indicate the activation of the intrinsic pathway of apoptosis due to caspase 3 detection. Caspase 8 in HeLa had no change, corresponding to the data obtained in the microarray (**Figure 3A**). Contrarily, in SiHa an increase in caspase-8 detection is maintained from 8 to 24 h, whereas in CaSki, an increase in caspase-8 is observed up to 12 h and decreases at 24 h. Likewise, a rise of p21 in HeLa is observed (**Figure 2**). The detection of XIAP in the three cell lines decreases as the exposure time with TT expires, emphasizing the absence in HeLa and SiHa at 24 h. Detection of BAX and BAK increased in the first hours of stimulation, although detection decreased after 24 h.

These findings together with the microarray assay and the Western blotting are complementary to each other. Information validates the proapoptotic proteins activated by the TT through caspase 3.

### 3.3 Early and late apoptoses are stimulated by the TT

**Figure 4:**
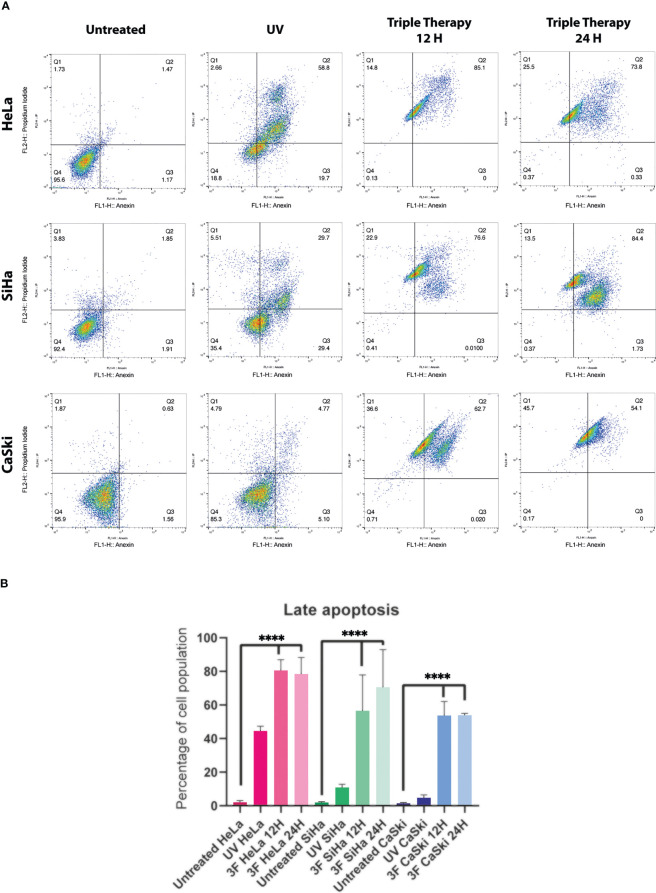
Dot plots from flow cytometry of Cervical cancer cells untreated, or treated with UV, or TT at 12 and 24 h of stimulation. **(A)** The dot plots are a representative image of at least three independent experiments. **(B)** The bar graph shows the percentage of cells undergoing apoptosis in response to triple therapy for 12 and 24 (h) *P* values were determined by ANOVA test; ****P ≤ 0.0001.

The death of cervical cancer cells through the apoptotic pathway was analyzed by flow cytometry using the Annexin V/IP kit and the IC_50_ values previously mentioned. The TT produced late apoptosis in the three CC cell lines, above 60%, observed in the dot plots (**Figure 4**, right columns) compared with controls. In HeLa, the percentage of cells in apoptosis was 85.1% and 73.8% in 12 and 24 h, respectively. In SiHa, the apoptosis population corresponded to 76.6% and 84.4% in 12 and 24 h, respectively, whereas in CaSki late apoptosis was observed in 62.7% in 12 h and 54.1% in 24 h. Apoptosis induction by UV radiation over 50% was observed only in HeLa.

In summary, the TT requires 12 h to induce apoptosis in more than 60% of the three CC cell lines, keeping high percentages until 24 h over the positive control.

### 3.4 Morphological analysis revealed apoptotic bodies and DNA fragmentation due to the TT

**Figure 5:**
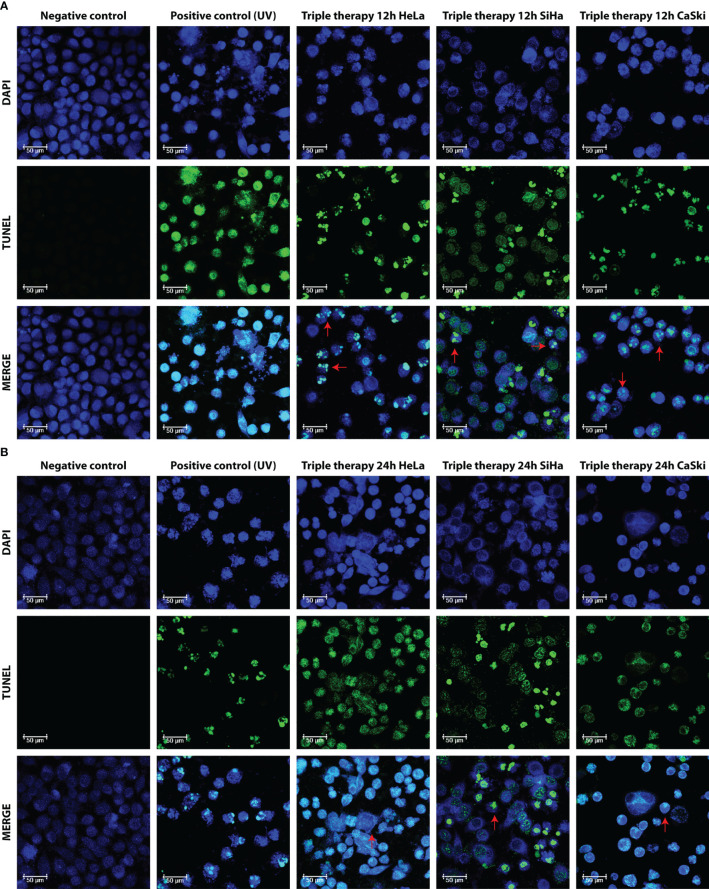
Morphological aspect of DNA fragmentation in HeLa, SiHa, and CaSki cells. Fluorescence images taken with LEICA confocal microscope at 63× with DAPI, TUNEL (dUTP cutoff marking of TUNEL deoxynucleotidyl transferase terminal), and MERGE markers in untreated cells (negative control), UV (positive control), and TT at 12 h **(A)** and 24 h **(B)** of exposure. Photos are representative of at least three independent experiments.

Apoptosis is complex and implies many morphological changes for the cell, for example, membrane permeability, chromatin condensation, and cell shrinkage. The DNA fragmentation produced by the TT was visualized by confocal microscopy using the TUNEL assay. **Figures 5A, B** show the images of the cells taken after 12 and 24 h of TT stimulus. The formation of micronuclei, small extranuclear bodies that originate from fragments of chromatids and/or chromosomes that are left behind in the anaphase of dividing cells and are not included in the main nucleus during the telophase, is observed in TUNEL and MERGE marked with green. Likewise, DNA condensation is observed at the periphery of the nuclear membrane, forming a growing structure, whereas in the negative control, no DNA fragmentation is observed (**Figure 5B** MERGE).

In **Figure 5B**, for the three cell lines and the positive control (UV) in TUNEL and MERGE pictures, observed in green, the formation of apoptotic bodies and detection of fragmented DNA throughout the cell, characteristics of late apoptosis, is observed. Results are in congruence with the Annexin V/IP assay.

### 3.5 Triggering of intrinsic apoptosis is required for inhibition of the mTOR signaling pathway by triple therapy in cervical cancer cells

**Figure 6:**
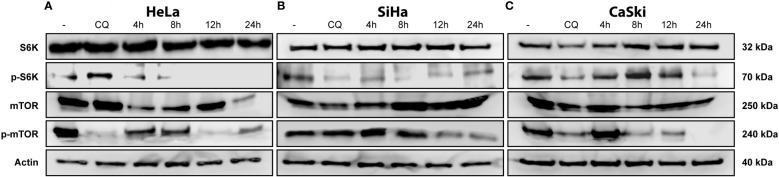
TT-induced suppression of the mTOR pathway. **(A)** HeLa, **(B)** SiHa, and **(C)** CaSki CC cell lines. All cells were treated with TT at 4, 8, 12, and 24 (h) Chloroquine (positive control). The blots are a representative figure of at least three independent experiments.

The master regulator, mTOR, plays a fundamental role in cell-cycle regulation, proliferation, apoptosis, and autophagy. To investigate the role of mTOR in triple therapy-induced apoptosis and autophagy, inhibition of mTOR and the substrate S6k was examined by Western blotting assay using phosphorylated antibodies. As shown in **Figure 6**, treatment with the triple therapy decreased the levels of phosphorylated mTOR and S6K in HeLa, SiHa, and CaSki cells. Our results displayed that the induction of intrinsic apoptosis through caspase-3 activation might be inhibiting the PI3K/AKT/mTOR signaling pathway. mTOR inhibition is an attractive therapeutic target in cancer due to its intervention in cellular functions such as cell survival or cell death (43), synthesis of biomolecules (44), and cell migration (45).

### 3.6 The TT suppressed the migration of cervical cancer cells *in vitro* at non-cytotoxic doses

**Figure 7:**
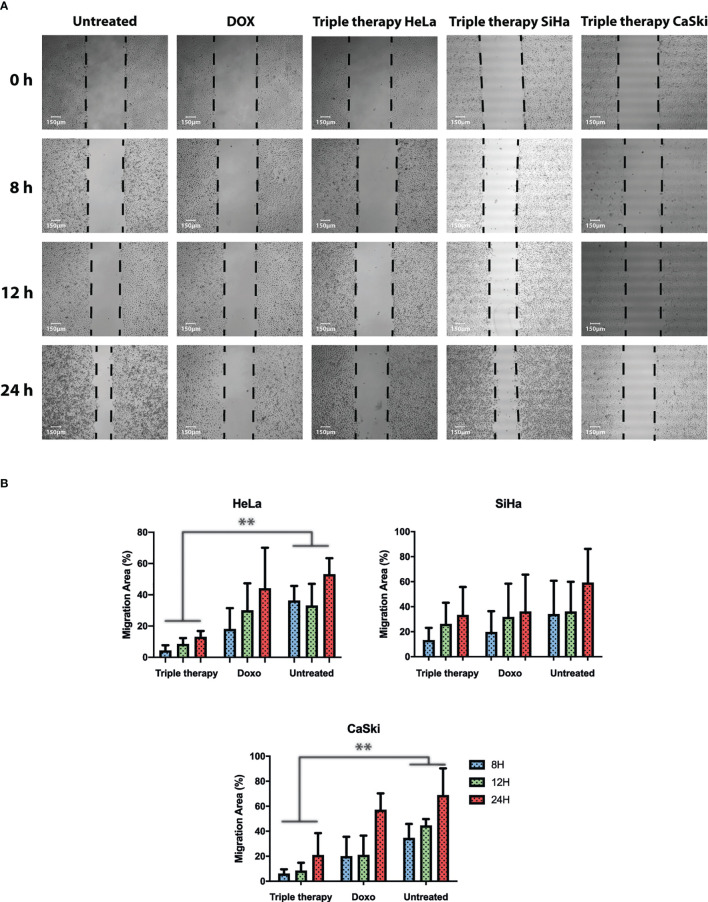
Triple therapy suppresses migration abilities in HeLa, SiHa, and CaSki cells compared with doxorubicin. A wound scratch assay was performed according to the procedure described in Materials and Methods in HeLa, SiHa, and CaSki. **(A)** Cell migration was observed at time 0 and 8, 12, and 24 h after scratching by photographs (magnification: 10×), and reduction of the initial scratch area was compared. **(B)** Quantitative analysis of the migration area was performed for HeLa, SiHa, and CaSki. All results are representative of three independent experiments. *P* values were determined by ANOVA test; **P ≤ 0.01.

Increasing evidence suggests that the mTOR pathway also plays a critical role in the regulation of cell migration. A wound healing assay was performed to test whether the triple therapy exerts an anti-migratory effect on cervical cancer cells. Cells were treated with the TT, doxorubicin, or untreated for 8, 12, and 24 h, respectively (**Figure 7**). Following treatment with the triple therapy for up to 24 h, cell migration was significantly inhibited, with a wound area of ~90.0% compared with the control of ~30% and doxorubicin in HeLa and CaSki ((**Figure 7B**). The control drug for this experiment was doxorubicin (positive control) to compare with the TT. These results suggest that the anti-migratory effects of the triple therapy may be potentially related to the induction of apoptosis and mTOR inhibition. In summary, the TT is a multitarget successful apoptosis-inducing combination.

## 4 Discussion

Cancer cells share several hallmarks that confer them a unique metabolism. However, the success of treatment not only depends on suppressing those characteristics but also on multifactorial events, such as sex, age, stage of disease, and response to treatment (43). The mentioned external factors establish different circumstances for treatment decisions turning the new therapeutic strategies into personal or individual medicine in the near future. In this context, our chemo design is optimistic. We propose a combination of three drugs targeting essential mechanisms of cell survival and proliferation, driving cancer cells to death in three different CC cell lines. Hela cells correspond to the HPV-18 form, whereas SiHa and CaSki correspond to the HPV-16 genotype (44).

The mTOR pathway has been explored as a potential therapeutic target in cancer as it integrates two of the main signals in the regulation of cell growth activated by tyrosine kinase receptors and nutrients, including amino acids and glucose (45). One of the principal pathways leading to mTOR activation is PI3K/AKT (46), which plays an essential role in cancer progression. mTOR phosphorylates S6K, its major effector leading to protein synthesis and promoting cell survival by inhibiting the pro-apoptotic protein BAD. Furthermore, mTOR regulates autophagy characterized by the formation of autophagic vesicles and the envelopment of organelles and proteins to recycle nutrients. Autophagy is activated mainly by the deprivation of nutrients such as glucose and amino acids. Therefore, the mTOR pathway is a sensor of the energy content of the cell, as it responds to AMP/ATP levels through AMPK, which inactivates mTOR when the AMP/ATP ratio increases. This event causes ULK1, ULK2, and Atg13 to be activated by phosphorylation leading to the initiation of autophagic vesicle conformation (47). Our findings indicate that TT involves the mTOR pathway to induce apoptosis in CC cell lines. Such an event is observed by the depletion of mTOR-activated levels when cells are stimulated with the TT (**Figure 6**, 12 h). According to these results and the previous ones obtained in colon and breast cancers, we can suggest that the depletion of mTOR is due to the synergistic interaction of the drug combination. In summary, metformin induces apoptosis through inhibition of mTOR *via* AMPK activation (36, 48), sodium oxamate inhibits the lactate dehydrogenase enzyme and aerobic glycolysis (49), and doxorubicin interferes with DNA synthesis (50, 51). Each drug eventually triggers key cell pathways leading to cell death, assembling a novel and attractive therapeutic strategy for cancer treatment.

The mechanism of action of these drugs is based on the inhibition of the energy generation pathways of the tumor cell: glycolysis, the mTOR pathway, and DNA synthesis (29, 35, 39). Thus, the combined inhibition of the glycolytic pathway could lead to a complete depletion of cellular ATP and increment cell death (35, 45–49, 52). Metformin-induced activation of AMPK is associated with increased oxidative stress, cell-cycle arrest, and induction of apoptosis (53). The combination of phenformin (another biguanide) and oxamate increases the number of cells in late apoptosis in different cancer cells and increases ROS levels and DNA damage (35, 36, 45, 47–49). The mix of metformin and sodium oxamate induces cell death in 85% of cells in late apoptosis in melanoma cancer, (54) and there was a decrease in LDHA, lactate, and ATP levels (55). Our group (50) showed metformin and oxamate plus doxorubicin-induced late apoptosis, an increase of protein caspase-3, and a drop in PARP-1 in triple-negative breast cancer cells. In this study, we aimed to identify the apoptosis pathway activated by the three drugs in combination in cervical cancer. For this purpose, we performed flow cytometry, Western blot, and confocal microscopy as complementary techniques to settle the activation of apoptosis. Our results indicate that the TT induced late apoptosis (**Figure 3**).

The execution of intrinsic apoptosis is characterized by permeabilization of the mitochondrial outer membrane (MOMP), enabling the release of cytochrome C leading to apoptosome formation and subsequent cleavage and activation of effector caspases (3 and 7) (56); as observed with the triple-therapy treatment, there is an increase in the detection of cleaved caspase-3, but not cleavage caspase-8, which is involved in extrinsic apoptosis (57). Taken together, these data indicate that the triple therapy may trigger intrinsic apoptosis (**Figure 2**).

It was reported elsewhere that metformin (51) and oxamate (52, 58) induced the activation of proteins implicated in intrinsic apoptosis (59) (BAD, BAX, cytochrome-C, Apaf-1, caspase-9, caspase-3 and 7) and decreased antiapoptotic proteins such as Bcl-2 and XIAP. It has been demonstrated that in the presence of an apoptotic stimulus, mitochondrial survivin is released to the cytosol, (60) where it inhibits the activation of caspase-3 through its interaction with XIAP to continue proliferating (61); metformin decreases XIAP expression in colorectal cancer by STAT3 suppression (47).

The induction of early apoptosis is related to the activation of Bcl-2 family proteins, depolarization of mitochondria, and activation of caspases (62) and occurs approximately 30 min after the applied stimulus (63). In the case of late apoptosis, it occurs after caspase activation induces nuclear condensation and the formation of apoptotic bodies, in a time frame of 4 to 24 h depending on the stimulus (63). Our outcomes revealed that cells treated with the TT induce apoptosis by the intrinsic pathway *via* caspase 3. Based on our previous studies, this results in decreased glucose consumption, inhibition of cell growth, and proliferation signals by blocking the mTOR pathway, eventually inducing late apoptosis cell death (50, 64).

One strategy in the search for anticancer treatments is the use of drugs known to be utilized for other treatments whose mechanism of action is involved in some target of the tumor cell. This is known as drug repositioning (65, 66). Its advantages are its toxicological history, which helps in terms of safety, knowledge of collateral effects, effectiveness, and even reduction of costs. The success of the therapies will also depend on the multiple performances of the pharmacological proposal, in order to attack the tumor cell at several points simultaneously. Our research covers two main targets in cancer therapy, energy metabolism and the inhibition of cell proliferation, key steps to stop tumor growth, and subsequent invasion and metastasis.

The combination of Dox–Met–Ox is an excellent candidate for drug repositioning in cancer treatment. Together, they exerted *in vitro* apoptosis induction in cervix, colon, and breast cancer cell lines (50, 64). Moreover, their synergy showed tumor growth suppression and no accumulative or new detectable side effects *in vivo*. The drugs composing the TT are well characterized at therapeutically effective doses worthy of further study and go a step forward to clinical trials. All the backgrounds supporting the effectiveness and safety of metformin and oxamate, added to the metformin and doxorubicin success in the clinic, were taken together for our research group to propose them as a therapeutic option for a diverse of cancers.

## Data availability statement

The original contributions presented in the study are included in the article/supplementary material. Further inquiries can be directed to the corresponding authors.

## Author contributions

ID-W performed and analyzed *in vitro* experiments and participated in the discussion of the article. SS-A performed the microscopy experiments and analyzed results. CC, JP, IA and VG-C analyzed the results and NJ-H and CP-P were responsible for conceptualization, discussion, writing, and funding resources. All authors contributed to the article and approved the submitted version.
